# Brain plasticity and sensorimotor deterioration as a function of 70 days head down tilt bed rest

**DOI:** 10.1371/journal.pone.0182236

**Published:** 2017-08-02

**Authors:** Vincent Koppelmans, Jacob J. Bloomberg, Yiri E. De Dios, Scott J. Wood, Patricia A. Reuter-Lorenz, Igor S. Kofman, Roy Riascos, Ajitkumar P. Mulavara, Rachael D. Seidler

**Affiliations:** 1 School of Kinesiology, University of Michigan, Ann Arbor, Michigan, United States of America; 2 NASA Johnson Space Center, Houston, TX, United States of America; 3 KBRwyle, Houston, TX, United States of America; 4 Department of Psychology, University of Michigan, Ann Arbor, Michigan, United States of America; 5 The University of Texas Health Science Center, Houston, TX, United States of America; 6 Neuroscience Program, University of Michigan, Ann Arbor, Michigan, United States of America; Tokai University, JAPAN

## Abstract

**Background:**

Adverse effects of spaceflight on sensorimotor function have been linked to altered somatosensory and vestibular inputs in the microgravity environment. Whether these spaceflight sequelae have a central nervous system component is unknown. However, experimental studies have shown spaceflight-induced brain structural changes in rodents’ sensorimotor brain regions. Understanding the neural correlates of spaceflight-related motor performance changes is important to ultimately develop tailored countermeasures that ensure mission success and astronauts’ health.

**Method:**

Head down-tilt bed rest (HDBR) can serve as a microgravity analog because it mimics body unloading and headward fluid shifts of microgravity. We conducted a 70-day 6° HDBR study with 18 right-handed males to investigate how microgravity affects focal gray matter (GM) brain volume. MRI data were collected at 7 time points before, during and post-HDBR. Standing balance and functional mobility were measured pre and post-HDBR. The same metrics were obtained at 4 time points over ~90 days from 12 control subjects, serving as reference data.

**Results:**

HDBR resulted in widespread increases GM in posterior parietal regions and decreases in frontal areas; recovery was not yet complete by 12 days post-HDBR. Additionally, HDBR led to balance and locomotor performance declines. Increases in a cluster comprising the precuneus, precentral and postcentral gyrus GM correlated with less deterioration or even improvement in standing balance. This association did not survive Bonferroni correction and should therefore be interpreted with caution. No brain or behavior changes were observed in control subjects.

**Conclusions:**

Our results parallel the sensorimotor deficits that astronauts experience post-flight. The widespread GM changes could reflect fluid redistribution. Additionally, the association between focal GM increase and balance changes suggests that HDBR also may result in neuroplastic adaptation. Future studies are warranted to determine causality and underlying mechanisms.

## Introduction

Previous studies have reported that long-duration spaceflight can adversely affect sensorimotor functioning. For example, astronauts performed worse on tests of locomotor functioning [1–4] and postural stability [2, 5] directly after a 6-month spaceflight mission in comparison to their pre-flight performance. Sensorimotor function at least partially recovers from days [3] to weeks [4] after return to Earth. Several factors account for the effects of spaceflight on sensorimotor functioning, including body unloading, altered vestibular inputs [6], and maladaptive reinterpretation of graviceptor inputs [7]. The neural changes linked to these effects have received less attention. Despite calls for neuroimaging studies already in the late 1990s [8], no prospective controlled studies have currently looked at the effects of spaceflight on human brain structure, and to what extent any potential changes could reflect compensatory neuroplasticity versus atrophy associated with radiation, stress, sleep loss, fluid redistribution, or other factors. Experience-dependent neuroplasticity takes place with skill learning across the lifespan [9–11]. It is thought that cellular and molecular level changes result in structural effects detectable with MRI [11]. It is plausible that similar brain structural changes take place in astronauts as they adapt their motor control to the microgravity environment. Rodent research has demonstrated that spaceflight can lead to structural brain gray matter (GM) alterations, including changes in the number and morphology of cortical synapses [12], changes in the distribution of axonal terminal type in the somatosensory cortex [13], and plasticity of cerebellar Purkinje cells and degeneration of Purkinje cells’ dendrites [14].

The exact mechanisms behind these brain structural alterations are unknown, though microgravity is a prominent factor that has been linked to several sequelae of spaceflight on the CNS [15]. In humans, long- duration head down tilt bed rest (HDBR) can serve as an analog environment to study the specific effects of prolonged axial body unloading and cephalad fluid shift on the sensorimotor system in isolation from the other physiological effects produced by exposure to the microgravity environment of spaceflight [16]. Several reports have shown that HDBR is associated with sensorimotor dysfunction (e.g., postural instability [17] and impaired functional mobility [16]) and altered cortical activation patterns [18–20]. To date, two studies have reported on structural brain changes in the human brain with HBDR. Roberts and colleagues have reported brain tissue expansion in the central frontoparietal regions and contraction in orbitofrontal regions in 8 subjects who underwent HDBR for 42 to 60 days [21]. Regions that showed expansion included the pre- and post-central gyri, which receive tactile input and are important for motor execution. Li et al. reported small GM increases in posterior parietal regions and around the falx cerebri, as well as decreases in GM in frontal brain regions in 18 subjects who completed 30 days of HDBR [22]. The latter study also suggested white matter alterations as a function of HDBR, but these results were only seen using a very lenient statistical threshold (*p*<0.01 uncorrected for multiple comparisons). Additionally, a study in rodents has revealed that hindlimb unloading leads to structural changes in the somatosensory cortex [23]. Another study reported alterations of glial cells in neuronal cell cultures that were exposed to simulated microgravity [24]. Together, these studies suggest that HDBR is associated with structural brain changes. However, to date no studies in humans have looked at multiple time points, which allows investigation of the temporal dynamics of changes with HDBR. Moreover, none of the structural MRI HDBR studies so far have included a control group, making it difficult to interpret results. Finally, no studies have examined associations between HDBR-induced brain and behavioral changes to assess whether they are related. In consideration of the health and functional performance of astronauts during and post-flight, it is important to characterize the extent, longevity, and neural bases of changes in sensorimotor behavior that are induced by a spaceflight analog environment. Identifying the underlying mechanisms of these changes is a necessary step towards developing countermeasures that could help minimize the deleterious effects of spaceflight and accelerate functional sensorimotor recovery of astronauts post-flight. To explore the effect of HDBR on sensorimotor performance, brain GM morphology, and their interaction, we conducted a prospective longitudinal 70-day 6-degrees HDBR study with 18 right-handed males. Effects of HDBR were compared to changes over time in 12 male control subjects who were assessed 4 times over a similar time course using the same protocol [15]. Pre, during and post-HDBR sensorimotor performance was assessed and structural MRI scans were obtained.

Based on previous HDBR studies and the reported effects of spaceflight and microgravity on sensorimotor and vestibular function, we hypothesized that HDBR would adversely affect brain regions involved in balance, perception, and motor control. We therefore specifically focused on the following regions that are involved in gait and balance performance: the primary motor cortex (control and execution of voluntary movement), the somatosensory cortex (main sensory receptive area for touch), the vestibular cortex (spatial orientation including balance and self-motion perception), and the cerebellum (sensorimotor coordination). We hypothesized that decreases in brain structure would correlate with sensorimotor performance deterioration. Furthermore, based on previous behavioral bed rest studies we expected that HDBR induced behavioral dysfunction and therefore also structural brain changes would at least partially recover during the post HDBR re-adaptation phase. Although the current study is tailored to investigate the effects of a spaceflight analog environment on brain structure and motor function, results from this study also have significant implications for those on extended bed rest due to clinical conditions.

## 2. Materials and methods

### 2.1 Study design

**Fig 1:**
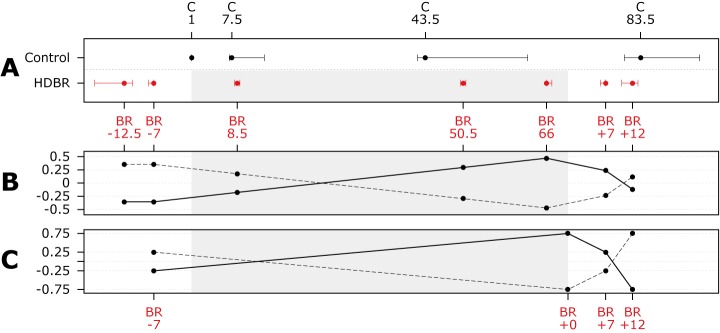
Time line and statistical contrasts. **A)** Scaled time line of data collection points for control and HDBR subjects. Days are median assessment days for all subjects with interquartile ranges. The gray rectangle indicates time in BR. (HD)BR = head down tilt bed rest; e.g. BR-12 = 12 days pre HDBR; C1 = first assessment day for control subjects.; **B)** Linear mixed model contrast to test for effects of HDBR on focal gray matter. solid line = contrast assuming stable gray matter volume pre-HDBR, linear increase in gray matter over HDBR, and partial recovery post-HDBR; dashed line = contrast assuming stable gray matter volume pre-HDBR, linear decrease in gray matter over HDBR, and partial recovery post-HDBR; **C)** Linear mixed model contrast to test for effects of HDBR on behavior. solid line = contrast assuming increase in time needed to complete the functional mobility test (i.e., performance decrease) from pre-HDBR to post-HDBR, with recovery post-HDBR; dashed line = contrast assuming decrease in standing balance performance (i.e., SOT 5) from pre-HDBR to post-HDBR, with recovery post-HDBR.

The full protocol of our study has been published previously [15]. This is a prospective longitudinal 6° HDBR study with 18 subjects randomized to two HDBR groups (HDBR exercise subjects and HDBR control subjects: see §2.2). For all subjects, MRI data was collected at 7 time points: pre- (-12.5 and -7 days), during (8.5, 50.5 and 66 days) and post- (+7 and + 12 days) HDBR whereas sensorimotor behavioral measures were obtained pre- (-12.5 and -7 days) and post- (+0, +7 and + 12 days) HDBR (see Fig 1A).

All HDBR subjects participated in a 70-day, 6°-HDBR experiment conducted at the bed rest facility located at the University of Texas Medical Branch (Galveston, TX). The bed rest program is a framework designed by NASA that offers the possibility to study HDBR as an experimental analog for spaceflight. Subjects who agreed and qualified to participate were admitted 13–22 days before going into HDBR and were released 12 days post-HDBR.

To be able to interpret effects of HDBR on brain structure and sensorimotor performance with regard to practice effects and random variation we used data from a separate study in 12 healthy control subjects who completed the exact same measurements as the HDBR subjects at four different time points over 90 days (i.e., day 1, 7.5, 43.5 and 83.5; see Fig 1A).

The HDBR and control assessment time points did not line up because the testing timeline of the control subjects was optimized for another study (see [15]).

### 2.2 Participants

All HDBR participants were males with mean age of 31.1 ± 4.7 years at time of admission (range: 25.7–39.8 years). Control subjects were males with mean age of 41.4 ± 9.9 years at time of first assessment (range 26.2–59.7). Control subjects were recruited from the NASA Johnson Space Center (JSC) subject pool, which consists of JSC employees. They went about their daily lives between test sessions. Sixteen out of the 18 HDBR subjects (89%) and 10 out of 12 control subjects (83%) were right handed (no group difference, odds ratio = 0.29, se = 0.38). Performance on tests of spatial processing (i.e., Thurstone’s 2D card mental rotation test and a 3D cube mental rotation test) [25] was not significantly different between HDBR and control subjects at baseline (data not shown).

To qualify for the study, subjects needed to pass an Air Force Class III equivalent physical examination.

This study was conducted in compliance with the Code of Ethics of the World Medical Association (Declaration of Helsinki) and approved by the institutional review boards of the University of Michigan, the University of Texas Medical Branch (UTMB), and NASA Johnson Space Center. Written informed consent was obtained from all participants. Both HDBR subjects and control subjects received monetary compensation for their participation.

#### 2.2.1 Bed rest intervention

Participants remained in the HDBR position at all times with the exception of 10 minutes during each meal, when they were allowed to prop up their head with their hand while eating. Participants were medically monitored and under diet control to maintain constant body weight throughout the HDBR period.

#### 2.2.2 Exercise intervention

In the framework of a different study on the effects of HDBR on physical fitness, subjects were randomly assigned to either an HDBR control group (n = 5) or one of two HDBR exercise groups (n = 5 and n = 8). Both HDBR exercise groups completed the same intensity exercise, but differed in the equipment that was used. Exercise participants started exercising 20 days before the start of HDBR; the first ~20 days of exercise included orientation to supine exercise and a gradual increase in intensity. The full exercise program began with the start of HDBR. Here, we will report on the effects of HDBR on brain function and motor behavior pooled over all subjects (i.e., HDBR exercise + HDBR control subjects) to have optimal power to detect effects of HDBR on central nervous system function and structure. To focus on the effects of HDBR we adjusted for any potential effects of exercise in our analyses.

### 2.3 Procedure

#### 2.3.1 Image acquisition

For the HDBR subjects imaging data were collected using a 3-Tesla Siemens Magnetom Skyra MRI scanner utilizing a 32 channel head coil. We used a T1-weighted gradient-echo pulse sequence (Magnetization Prepared Rapid Gradient Echo; MPRAGE) with the following parameters: 3D T1 sagittal overlay (TR = 1900 ms, TE = 2.44 ms, flip angle = 9°, FOV = 270×270 mm, slice thickness = 0.9 mm, 192 slices, matrix = 288×288, voxel size = 0.94×0.94×0.90 = 0.80 mm^3^). In 6 of our 18 subjects scan parameters were unintentionally altered slightly at up to two time points. These alterations were very minor (e.g., the voxel resolution changed from 0.94mm to 0.98mm) and were not systematically distributed over the time points regarding the intervention and recovery time course. Removing these scans did not significantly change our results. For one subject at one time point MRI scans were lost during data transfer.

For the control subjects imaging data were 3-Tesla Siemens Magnetom Verio utilizing a 32 channel head coil. We used a T1-weighted gradient-echo pulse sequence with the following parameters: 3D T1 axial overlay (TR = 1900 ms, TE = 2.32 ms, flip angle = 9°, FOV = 250×250 mm, slice thickness = 0.9 mm, 192 slices, matrix = 512×512, voxel size = 0.49×0.49×0.90 = 0.22 mm^3^). For pre-processing, the in-plane resolution was down sampled to 0.94x0.94mm. For two subjects, data from one time point were unavailable.

#### 2.3.2 Image preprocessing

The following software packages were used for image analysis: FMRIB Software Library (FSL) version 5.0.4; the Voxel Based Morphometry 8 (VBM8) v435 toolbox [26] for Statistical Parametric Mapping (SPM) 8 v4667, running under MATLAB 7.14.0.739 (R2012a); and NeuroAnalytica (formerly Brain Research: Analysis of Images Networks and Systems; BRAINS) [27, 28].

Field inhomogeneity estimation and correction were applied to all T1 images within a subject specific brain mask using N4ITK [29]. The brain masks were created using FSL’s Brain Extraction Tool (BET) [30] with robust brain center estimation and a fractional intensity threshold of 0.15.

#### 2.3.3 Regional GM volume

Gray matter (GM) volume was measured in five brain regions of interest that play important roles in motor execution (primary motor cortex), sensory perception (somatosensory cortex and cerebellum lobule VIIIb), balance (vestibular cortex), and motor coordination (cerebellum lobule V), [31, 32]. Left primary motor cortex and left somatosensory cortex masks from the International Consortium for Brain Mapping atlas labels were used to obtain GM from the smoothed normalized GM images that were processed as part of the voxel based morphometry 8 toolbox (VBM8; see §2.4.4) [26]. A spherical region of interest around Montreal Neurological Institute (MNI) coordinate = 42,-24,18 with a diameter of 10mm (264mm^3^) was used to obtain gray matter volume measures of the right vestibular cortex. The MNI coordinate was selected on the basis of a meta-analysis that located operculum parietale 2 (i.e., the homologue of the parietoinsular vestibular cortex in nonhuman primates) at this location [33, 34]. Right lobule V and right lobule VIIIb of the cerebellum were masked out from the smoothed normalized VBM8 GM images using masks obtained from the SUIT cerebellum atlas [35]. NeuroAnalytica/BRAINS software was used to automatically obtain total intracranial volume measures from our T1-weighted images [27, 28]. For data interpretation purposes we additionally tested if there were changes in total brain volume (TBV). TBV was defined as total GM volume + total white matter volume. Total white matter volume was automatically obtained from the VBM8 pipeline.

#### 2.3.4 Focal GM volume

Longitudinal voxel based morphometry using the VBM8 pipeline was used to assess changes in focal GM volume over time in HDBR and control subjects. The pipeline iterates over subjects’ image sets. For each subject all images were rigidly registered to the subject specific mean image. Spatial normalization parameters to bring the images into MNI standard space were calculated from the segmented mean image and applied to the GM tissue class of all time points per subject. In a final step, all normalized segmentations were once more realigned and then smoothed with an 8 mm full-width at half-maximum Gaussian kernel to increase signal to noise ratio [26].

#### 2.3.5 Reliability of GM volume measures

To draw inferences from longitudinal data it is important that the data are reliable and not subject to random variation over time. We used Stata’s (Stata SE v13.1, StataCorp, Texas) post-estimation (estat icc) command to calculate the intra-class correlation coefficient (ICC) of the volumes of the Regions of Interest (ROI) between the two baseline measures of the HDBR subjects (i.e., BR -12.5 and BR -7) and between the first two measures of the control subjects (i.e., assessment day 1 and assessment day 7.5). The ICC metric ranges from 0 (no agreement) to 1 (perfect agreement). ROIs with ICC values below 0.5 were excluded from further analysis because of insufficient reliability. In addition, we used the ICC toolbox for SPM [36] to obtain the consistent agreement of gray matter maps from 12 days pre-HDBR to 8-days pre-HDBR [37].

#### 2.3.6 Functional mobility and balance performance

Motor behavioral outcomes of our HDBR study have been published previously [25]. Here we report on the motor behavioral effects of HDBR in relation to structural brain changes. We measured posture control with a computerized dynamic posturography system (Equitest, NeuroCom International, Clackamas, OR) [16] and the in-house developed Functional Mobility Test (FMT) [4]. Both tests have been described in full previously [15]. The FMT is an obstacle course that subjects have to pass as quickly and safely as possible without touching any of the obstacles [4]. Obstacles include foam bars, slalom pylons, and hurdles. The first part of the course is set up on a concrete floor while the second part of the course is constructed on medium density foam, which makes proprioceptive input unreliable. Outcome measures of the FMT are times needed to finish the first half of the course, the second half of the course, and the total course completion time.

Balance control was measured using the Sensory Organization Tests (SOTs) provided by EquiTest System platform (NeuroCom, Clackamas, OR) [38]. During testing, subjects were instructed to maintain stable upright posture for three 20-second trials per condition with feet positioned shoulder width apart, eyes closed and arms folded across the chest. All trials were conducted with a sway-referenced support surface intended to disrupt somatosensory feedback and therefore reflect how well vestibular input could be utilized to maintain balance. The center of pressure in both anterior-posterior (AP) and medial-lateral (ML) directions was obtained from the force plate and then filtered to estimate center of mass (COM). The subject’s sway angle was then derived from the COM that was assumed to be above the support surface at approximately 55% of total height [39]. The AP peak-to-peak sway angle was used to compute a continuous equilibrium (cEQ) score scaled relative to a maximum theoretical peak-to-peak sway of 12.5 and normalized by the percentage of the trial completed (referred to as SOT-5). Each trial was repeated three times. In addition to SOT-5, subjects completed the task once more while they pitched their heads ±20° at 0.33 Hz as cued by an oscillating tone provided over headphones (referred to as SOT-5M). SOT-5M is more difficult than the SOT-5 by requiring voluntary head movements and thus integration of both semicircular canal (angular) and otolith (linear) cues.

#### 2.3.7 Analysis

**2.3.7.1 Regional GM volume.** Inspection of the data showed that all regional volumes were normally distributed. Volumes were expressed as proportion of ICV to adjust for head size. This is a conventional way of standardizing brain volumes (see [40, 41]). We used linear mixed models (LMM) to a) compare changes over time between HDBR and control subjects; and b) model the recovery time course and stepwise changes in GM volume within HDBR subjects:

1. We compared the slope from pre-HDBR to the end of HDBR (i.e., time points BR -7, 8.5, 50.5 and 66) to the slope of the control subjects (all 4 time points). We only included these four HDBR assessments because we wanted to test whether there are either increases/decreases over the course of bed rest relative to control subjects. Random intercepts were modeled for subjects. Time was included as a *continuous* variable and age as a confounder.
2. We used pre-specified contrast weights that model a stable baseline, increases/decreases with HDBR, and recovery post-HDBR (see Fig 1B). Random intercepts were modeled for subjects. Time was included as a *factor* variable.

Restricted maximum likelihood (REML) was applied in the LMM because REML variance components are less biased in small samples [42]. To adjust for potential effects of exercise on GM volume we entered the HDBR group (exercise and non-exercise) and the group-by-time interaction terms in our model. Bonferroni correction for multiple testing was used unless stated otherwise.

**2.3.7.2 Focal GM volume.** To test the effects of HDBR on focal GM changes we a) compared focal GM changes over time between HDBR and control subjects; and b) we modeled focal GM changes over time within HDBR using pre-specified contrast weights (see Fig 1B). Recently, concern was raised regarding inflation of false positives for cluster-wise inference with parametric models [43]. Therefore we used non-parametric permutation tests for inference whenever possible. All voxel-wise analyses were family-wise error corrected (*p*<0.05) and Bonferroni adjusted for contrasts tested.

1. To be able to directly compare focal changes over time between the two groups we calculated percentage signal change maps for each group using linear regression. For HDBR subjects we calculated the slope over BR -7, 8.5, 50.5 and 66. For control subjects we used all four time points. The slope was then expressed as a percentage of the intercept to take into account baseline differences. We used a non-parametric voxel-wise two-sample t-test implemented in FSL’s ‘randomise’ [44] with age as a covariate and 15,000 random permutations to test for focal differences in changes over time between the two groups.
2. We used an LMM with time as factor implemented in SPM [26] to test the effects of HDBR including the recovery time course. For this, we used pre-specified contrast weights (see Fig 1B). To adjust for potential effects of exercise on gray matter volume or sensorimotor performance, we entered HDBR group (exercise and non-exercise) and the group-by-time interaction terms in our model. Additionally, within this model we conducted post-hoc pairwise comparisons between pre-HDBR and subsequent. Voxels with a value smaller than 0.1 were excluded from analysis to account for edge effects.

**2.3.7.3 Functional mobility and balance performance.** Similar LMM models as those used to test changes in regional GM volume (see §2.3.7.1) were used to test changes in gait and balance performance. Thus, we a) compared changes over time between HDBR and control subjects; and b) modeled the recovery time course and stepwise changes in performance within HDBR subjects:

1. We compared the slope from pre-HDBR to the end of HDBR (i.e., time points BR -7, and +0) to the slope of the control subjects over time points 7.5, 43.5, and 83.5. In this case the first time points of the HDBR and control group were omitted to account for initial practice effects.
2. We used pre-specified contrast weights that model a stable baseline, increases/decreases with HDBR, and recovery post-HDBR (see Fig 1C). The first time point was excluded to account for practice effects. We only tested contrasts for negative effects of HDBR on sensorimotor performance with subsequent recovery, as we did not expect performance to increase as a function of HDBR.

All LMMs were Bonferroni corrected, modeled using REML, and adjusted for exercise (see §2.3.7.1).

**2.3.7.4 Brain-behavioral relationships.** We determined whether HDBR-associated a) regional GM changes and b) focal GM changes correlated with behavioral changes:

1. We used linear regression analysis to test whether *regional* GM volume changes of our predefined sensorimotor brain regions were associated with changes in motor performance (FMT, SOT-5, and SOT-5M). Volume changes were defined as the difference between 7 days pre-HDBR to 66 days in-HDBR. Motor performance changes were defined as the difference between 7 days pre-HDBR and the first day post-HDBR.
2. We used voxel-wise nonparametric permutation based analysis to test whether *focal* GM volume changes were associated with changes in motor performance (FMT, SOT-5, and SOT-5M). For the permutation test we ran 15,000 random permutations and applied variance smoothing with sigma 3.39 (equivalent to 8 mm full-width at half-maximum) implemented in FSL’s ‘randomize’ [44]. Focal GM contrast maps were calculated by subtracting GM maps collected at HDBR -8 and HDBR ~70. The analyses were restricted (i.e., masked) to those regions in which we observed a significant GM increase or decrease from 7 days pre-HDBR to 66 days in-HDBR and were adjusted for total intracranial volume. All voxel-wise analyses were family-wise error corrected (*p*<0.05).

## 3. Results

### 3.1 Reliability of GM volume measures

Within HDBR subjects we calculated the ICC over a 5.5-day interval (i.e., from 12.5 days pre-HDBR to 7 days pre-HDBR). The ICC of cerebellum lobule VIII was below 0.5 and therefore this region was excluded from further analysis. The ICC of the intracranial volume (ICV) and remaining four ROIs ranged from 0.89 to 0.99 indicating excellent reliability. The median overall whole brain ICC of our GM maps collected 12.5 and 7 days pre-HDBR was 0.79, indicating excellent reliability [45].

Within control subjects we calculated the ICC over a 7.5 day interval (i.e., assessment at day 1 to assessment at day 7.5). The ICC of cerebellum lobule VIII was ~0.3 indicating poor reliability. The ICC of ICV and the remaining four ROIs ranged from 0.78 to 0.99 indicating excellent reliability. The median overall whole brain ICC of gray matter maps was 0.65, indicating good reliability [45].

### 3.2 Brain volumetric changes as a function of HDBR

**Fig 2:**
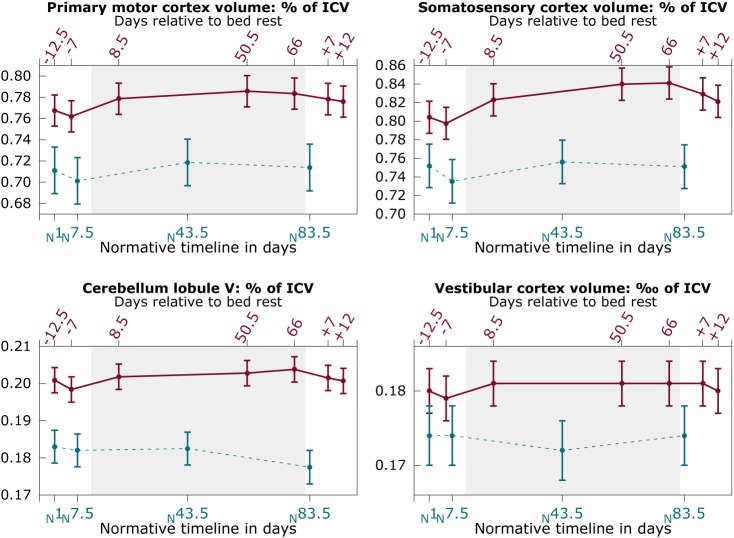
Regional gray matter changes as a function of bed rest. Solid red lines: HDBR subjects; Dashed blue lines: control subjects; Graphs show mean values over subjects with pooled standard errors. Y-axis shows volume of the region of interest as percentage of the intracranial volume (ICV), except for the vestibular cortex region (4^th^ panel), which shows volume as per mille of the ICV. Top x-axis shows the number of days relative to HDBR; bottom axis shows the time in days for control subjects relative to their first assessment.

Fig 2 shows the changes in GM volume over time for HDBR subjects and control subjects. HDBR subjects showed a significant increase in gray matter volume (% of ICV) over the course of HDBR in the somatosensory cortex (β_per day_ = 5.17×10^−4^ (% of ICV); SE = 1.6×10^−4^; *p* = .002) and cerebellum lobule V (β_per day_ = 1.26×10^−4^ (% of ICV); SE = 4.56×10^−5^; *p* = .006) relative to control subjects (see Fig 2). No significant changes over time were observed in HDBR subjects in the primary motor cortex or the vestibular cortex, or in any region in the control subjects.

Modeling the GM time course using our predefined contrast weights revealed a significant pattern of GM increases with HDBR and subsequent recovery in the primary motor cortex (χ^2^_(1,*N* = 18)_ = 24.43, *p* = < .001), somatosensory cortex (χ^2^_(1,*N* = 18)_ = 65.65, *p* = < .001), and cerebellum lobule V (χ^2^_(1,*N* = 18)_ = 16.14, *p* = < .001). No such effect was observed in the vestibular cortex. Post-hoc testing revealed that GM volume in the somatosensory cortex was larger at 7 days post-HDBR compared to pre-HDBR (β = 0.03 (% of ICV); SE = 0.01; *p* = .030). At 12 days post-HDBR, this difference was no longer significant.

### 3.3 Focal gray matter changes as a function of HDBR

**Fig 3:**
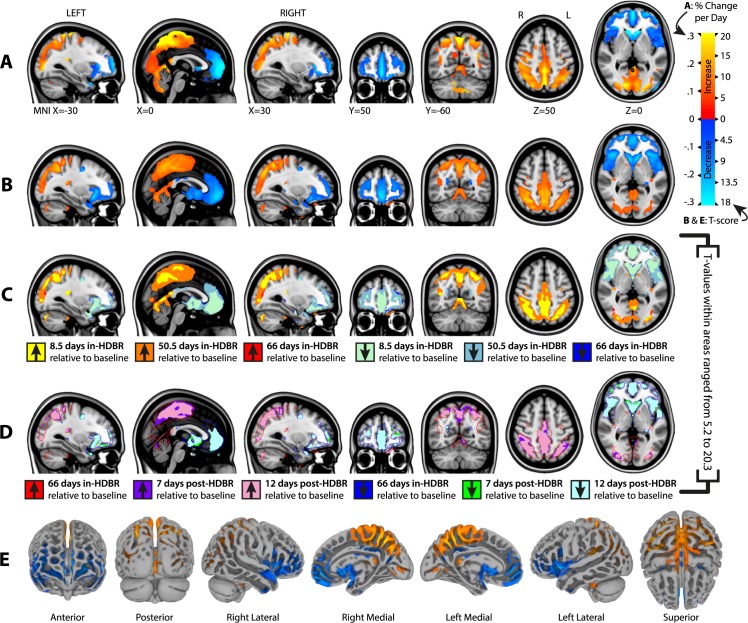
Focal gray matter changes as a function of bed rest. A) Percentage volume change from pre-HDBR to 66 days in HDBR tested against the percent volume change in control subjects over their 83.5 day time course; only areas with significant group-by-time interactions are shown. Gradients show the percentage change per day in gray matter volume in the HDBR subjects adjusted for the change in control subjects. B) Regions showing significant GM changes following predefined contrast weights (see Fig 1B) within HDBR subjects. Gradients show the distribution of T-values; C) Stepwise increases from pre-HDBR to 8.5, 50.5, and 66 days during HDBR; D) Stepwise differences with pre-HDBR at 7 and 12 days post-HDBR (i.e., recovery); E) Results shown in B) plotted on the MNI152 surface; MNI = Montreal Neurologic Institute 152 standard space.

Fig 3A shows areas with significant focal GM changes from pre-HDBR to the last assessment during HDBR relative to changes in control subjects. Large, widespread changes were seen in posterior parietal regions (GM increases) and fronto-temporal regions (GM decreases) (for an overview of anatomical locations see S1 Table). The average percentage increase in GM volume per voxel averaged across all voxels that exhibited significant increase was 0.16% per day in HDBR. The total GM increase from pre-HDBR to post-HDBR in this area was 27.8 ml. The average percentage decrease in GM volume per voxel averaged across all voxels that exhibited significant decrease was 0.14% per day in HDBR. The total GM decrease from pre-HDBR to post-HDBR in this area was 15.0 ml. No changes were observed in the control group. There were no significant differences in the rate of change in TBV between control subjects (on average 0.012% of ICV increase per day, se = 0.049, *p* = .81) and HDBR subjects (additional 0.005% of ICV increase, se = 0.095, *p* = .96).

Fig 3B and 3E show focal GM changes from pre-HDBR to post-HDBR modeled using the pre-specified contrast weights (for an overview of anatomical locations see S1 Table). The GM changes in Fig 3A and 3B are very similar, underlining the lack of change and reliability of GM in the control group.

Fig 3C and 3D show the results of the pairwise comparisons with the pre-HDBR GM maps. Post-hoc pairwise analysis of gray matter maps of HDBR subjects did not indicate differences between the two scans collected prior to HDBR. Therefore, baseline measures were pooled to obtain more robust pre-HDBR estimates. The pairwise comparisons showed that size of the regions in which we observed significant changes in GM increases and GM decreases gradually developed over the course of HDBR (Fig 3C) and gradually recovered post-HDBR (Fig 3D). However, the recovery of GM increase and GM decrease was incomplete at 12 days post-HDBR. S1 Table shows the corresponding anatomical names of the peak voxels within the regions showing significant GM changes per time point.

To obtain a better understanding of the potential relationship between GM increases and GM decreases with HDBR *a posteriori*, we correlated the time course of volume changes in posterior and anterior regions of the brain. For this, we created a mask from the largest cluster in which we found significant GM increases at 8.5 days in HDBR, and one from the largest cluster in which we found significant GM decreases at 8.5 days in HDBR. These masks were used to obtain GM volume per subject per time point from the smoothed GM images. Within each subject we correlated the GM time courses (all 7 time points) of the two regions using Spearman’s rank test because of non-normal distribution. The median correlation coefficient over all 18 subjects was -0.86 (interquartile range = -0.82 to -0.93) indicating that loss of GM in frontal areas was strongly correlated with increases in GM in posterior parietal regions.

### 3.4 Sensorimotor changes as a function of HDBR

**Fig 4:**
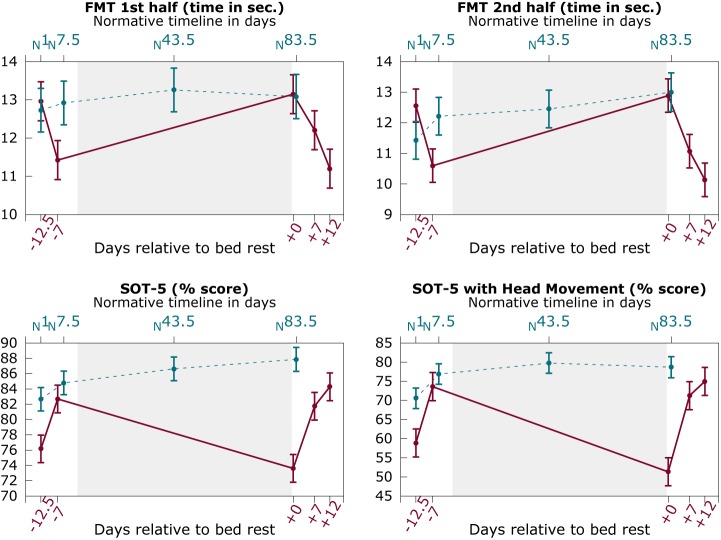
Sensorimotor performance changes as a function of bed rest. Solid red lines: HDBR subjects; Dashed blue lines: control subjects; Graphs show mean values over subjects with pooled standard errors. Y-axis shows time in seconds to complete the FMT (top two panels) or the percentage score of the SOT-5 (bottom two panels). Top x-axis shows the time in days for control subjects relative to their first assessment; bottom axis shows the number of days relative to HDBR; FMT = Functional Mobility Test; SOT-5 = Sensory Organization Test 5.

Fig 4 shows the changes in functional mobility and balance performance over time for HDBR subjects and control subjects. HDBR subjects showed a significant increase in the time in seconds needed to complete the total FMT (β_per day_ = 7.07×10^−2^; SE = 2.0×10^−2^; *p* < .001), the first half of the FMT (β_per day_ = 3.48×10^−2^; SE = 8.7×10^−3^; *p* < .001), the second half of the FMT (β_per day_ = 3.81×10^−2^; SE = 1.4×10^−2^; *p* = .006), SOT 5 balance performance (β_per day_ = -1.62×10^−1^; SE = 4.75×10^−2^; *p* = .001), and SOT 5M balance performance with head movement (β_per day_ = -4.24×10^−1^; SE = 1.10×10^−1^; *p* < .001) from pre-HDBR to post-HDBR relative to control subjects. Except for FMT 2^nd^ half, these results survived Bonferroni correction for alpha inflation. Our models did not demonstrate any significant performance changes over time in the control group.

Modeling behavioral changes using our predefined contrast weights revealed a significant pattern of sensorimotor decreases with HDBR and subsequent recovery post-HDBR for time to complete the total FMT (χ^2^_(1,*N* = 18)_ = 59.10, *p* < .001), the first half of the FMT (χ^2^_(1,*N* = 18)_ = 47.77, *p* < .001), the second half of the FMT (χ^2^_(1,*N* = 18)_ = 56.82, *p* < .001), and SOT 5 balance performance (χ^2^_(1,*N* = 18)_ = 18.60, *p* < .001), and SOT 5 balance performance with head movement (χ^2^_(1,*N* = 18)_ = 36.35, *p* < .001). Post-hoc tests indicated that at 8 days post-HDBR, the time in seconds needed to complete the total FMT (β = 4.25; SE = 1.17; *p* < .001), the first half of the FMT (β = 2.41; SE = 0.55; *p* < .001), and the second half of the FMT (β = 1.84; SE = 0.71; *p* = .009) were still significantly increased compared to performance at 8 days pre-HDBR. These differences were no longer significant at 12 days post-HDBR. SOT 5 balance performance was not significantly different post-HDBR compared to 8 days pre-HDBR.

### 3.5 Brain-behavioral relationships

Balance performance changes were positively associated with GM volume changes in the vestibular cortex (*b* = 6306.72, t(16) = 2.69, *p* = .017) and GM volume changes in cerebellar lobule V (*b* = 743.71, t(16) = 2.90, *p* = .011). Volume within these regions increased in some subjects and decreased in others. Larger volumetric decreases were associated with larger balance decrements, whereas larger volumetric increases were associated with smaller deterioration or even slight improvements in balance performance. These associations were no longer significant after correcting for multiple comparisons. No other significant associations were observed between changes in brain volume of our regions of interest and motor behavioral changes.

**Fig 5:**
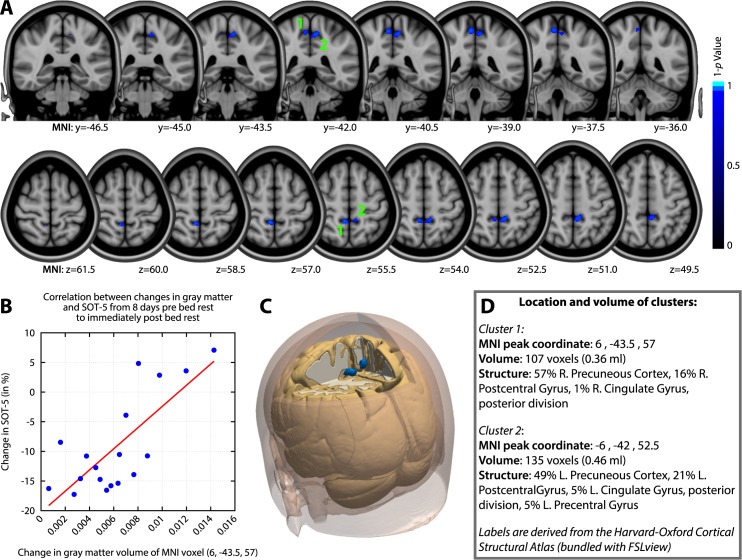
Associations between HDBR related gray matter changes and motor behavioral changes. A) Slices of the MNI brain depicting regions in which gray matter volumetric changes were significantly associated with changes in SOT-5 standing balance performance; Larger GM increases over time in blue areas were associated with better (i.e. smaller decreases or larger increases) standing balance performance. B) Scatterplot of the change in GM volume at the peak significant voxel and the change in balance performance; C) A 3D render of the locations of the two significant areas on the MNI brain. D) Summary of the volume and exact peak voxel coordinates of the two significant clusters. The Harvard-Oxford cortical structural atlas [46] was used for identification of the anatomical regions; MNI = Montreal Neurologic Institute.

Voxel-wise analysis revealed significant associations between focal GM increases from the last day pre-HDBR to the last day in-HDBR and changes in SOT-5 performance over the same time period (see Fig 5). The Pearson correlation coefficient of the peak voxel was .76. Subjects who presented with the largest GM increases in the bilateral precuneus and pre- and postcentral gyrus showed the smallest decrements or even improvements in standing balance (SOT-5) performance. No associations were observed between local GM changes and changes in FMT performance. These results were adjusted for family-wise error correction at the voxel-level. They did not survive additional Bonferroni (*p*<(0.05/4)) correction to adjust for the fact that we tested positive and negative correlations for 2 behavioral outcome measures (i.e., 4 tests).

## 4. Discussion

By comparing longitudinal data from 18 HDBR and 12 control subjects we show that 70 days of 6 degrees HDBR results in significant volumetric GM changes in brain structures that regulate planning, control and execution of voluntary movement, sensorimotor coordination, and that process sensory inputs. Furthermore, HDBR leads to deterioration in functional mobility and standing balance performance. Change in balance performance was associated with GM changes in a cluster that comprised the precuneus, pre- and postcentral gyri, although this result did not survive Bonferroni correction. These brain regions are respectively known for their role in sensory perception and motor control and their contributions to mental orientation in space, time and person [47]. In general, larger volumetric increases were associated with smaller declines in balance. HDBR could affect balance through compensatory reweighting of sensory information in response to body unloading [6]. Hence, one of the potential mechanisms that could explain the GM plasticity is as a response to the altered sensory inputs presented to subjects as a result of HDBR. Control subjects did not show GM changes over time and beyond their second measurement, no learning effects were observed in any of our behavioral measures.

The volumetric GM changes that were observed throughout the brain with HDBR could roughly be divided into posterior parietal GM increases and fronto-temporal decreases. Although the effects of HDBR on behavior and GM volume partially recovered after HDBR, the observed structural brain changes did not fully dissipate by 12 days post-HDBR.

Our neuroimaging measures proved to be reliable overall. The somewhat lower reliability of the voxelwise GM maps in control subjects compared to HDBR subjects could be the result of the longer interval between the two measurements (i.e., 5.5 days for HDBR subjects and 7.5 days for control subjects), although both were ‘good’ or better.

GM changes can reflect multiple processes that interact dynamically over time. Segmentation of GM in our study was based on location and/or relative voxel intensity. Conventional T1-weighted MRI scans do not offer the resolution to look at changes on the cellular level and therefore we can only speculate about the mechanisms behind the GM volumetric changes that we observed [11, 48]. We first return to our hypothesis that brain changes might correlate with behavioral changes, suggesting either atrophy or compensatory neuroplasticity depending on the direction of associations. Subsequently we discuss brain fluid redistribution as another potential mechanism.

### 4.1 Gray matter plasticity

The GM volumetric changes we observed could represent positive and negative neuroplastic effects [49]. Posterior parietal regions play an important role in spatial attention, body orientation, and sensorimotor integration [50] whereas several regions in the frontal and cerebellar cortex are crucial for motor control [51]. Structural changes in these regions could therefore potentially affect the functions regulated by these regions. Indeed, we observed that the GM increases in a cluster comprising parts of the precuneus (a region involved in controlling spatial aspects of motor behavior and motor imagery [52]), and the pre- and postcentral gyri correlated with less reduction in postural equilibrium scores, although these results did not survive Bonferroni correction and should therefore be interpreted with caution. Subjects with the largest GM increases showed the least drop or even slight improvements in postural equilibrium. This could mean that GM plasticity in this region reflects adaptive responses to the changed body orientation similar to that seen in balance performance post-HDBR. Axon sprouting, dendritic branching and synaptogenesis, changes in glial number and morphology, and angiogenesis are structural changes known to take place in the adult human brain. They therefore may explain the here observed GM changes and their relation to function (for an overview, see: [11]). Several experimental studies showed that recent sensory experiences can result in structural changes in the somatosensory cortex [53]. Thus, reduced somatosensory input from body unloading in HDBR could have affected cortical plasticity. This is in line with a review on the effects of microgravity on mental imagery that concluded that microgravity impairs spatial updating of a mental representation of one’s own body or body-part [54]. Finally, previous research showed that reducing somatosensory input can make other sensory inputs (e.g. vision and vestibular input) more responsive [6]. Integrative changes in sensory processing could explain the GM changes and potentially result in functional connectivity changes in motor brain regions, such as we reported previously [55]. Further evidence for brain plasticity changes in HDBR come from a previous functional MRI study in which we observed increased recruitment of frontal, parietal and cerebellar regions with HDBR during a cognitively demanding dual task [56].

Even though the significant GM changes occurred already at 8.5 days in HDBR, they could reflect neuroplasticity. This interpretation is consistent with a previous prospective voxel based morphometry study investigating brain changes over five days of repeating a sequential pinch force task for 20 minutes[57]. This intervention resulted in GM changes in the primary motor cortex and the premotor cortex, which furthermore correlated with performance improvements. Another longitudinal study revealed cortical thickness changes after only 16 days of hand immobility [58]. In this study immobilization of the right arm in 10 right-handed individuals resulted in decreases in the left primary motor and somatosensory cortical thickness. In addition, increased motor skill of the left hand together with cortical thickness increases of the right motor cortex were reported.

### 4.2 ICP related headward fluid shift

An alternative explanation for the GM increases and decreases are local changes in intracranial interstitial fluid [48, 59]. A change in posture from the upright to supine position elevates the intracranial pressure because of a decrease in venous outflow, an increase in venous pressure, and an increase in intracranial CSF volume [60, 61]. Experimental studies have shown that increased CSF pressure could lead to the CSF flowing from the ventricles and subarachnoid space into the extracellular space [62] and some suggest that head down-tilt could produce brain edema [61]. The inflow of CSF into the interstitial fluid could present as increased GM volume on T1-weighted MRI scans. In contrast, GM decreases could reflect interstitial CSF outflow.

A previous MRI study by Caprihan et al. that measured changes in water content in five ROI in five subjects using T2-weighted imaging showed that a head down-tilt position, in comparison to the supine position, can lead to increases in CSF in the midsagittal posterior parietal subarachnoid compartment [63]. In this region we found an increase in GM volume. However, the authors did not report decreases in fluid content in GM volume. This could be due to the fact that none of the ROIs in their study overlapped with any of the regions in which we observed GM volumetric decreases. Future studies should incorporate measures of intracranial pressure or for example high resolution MR images of the ventricles to investigate whether increased ICP is a causal factor of structural brain changes in HDBR.

### 4.3 Rotated posture induced redistribution of fluid

A related explanation could be that the rotated posture results in anterior-to-posterior fluid redistribution within the intracranial compartment. Thus, prolonged changes in gravitational direction may result in fluid redistribution in addition to the well described inferior to superior headward fluid shift. An MRI study evaluating CSF distribution in supine, sideways, and prone positions showed that subarachnoid CSF volume decreases on the side of the head closest to the ground [64]. This is potentially caused by a downward shift of brain tissue. Perhaps, gravity-related subarachnoid and interstitial CSF outflow in the posterior part of the brain could present as increased GM density. The opposite could be true for the frontal part of the brain where we observed a GM decrease. Even though our HDBR participants were allowed to roll over to their side or a prone position, we assume that they spent the majority of their time in bed lying on their back, allowing such fluid redistribution to occur. The strong proportionality that we observed between frontal GM loss and posterior parietal GM increases are indicative of fluid redistribution in HDBR. Moreover, the shift is in the expected direction when considering the body orientation in 6-degree HDBR. However, if this were the prime factor contributing to our results, we would expect to see more extensive increases in occipital lobe GM. VBM studies evaluating HDBR effects at different tilt angles would be required to test this hypothesis. Of further note is that not all HDBR studies provide pillows for their subjects, and therefore the exact pattern of redistribution between HDBR studies can slightly vary.

### 4.4 Relation to previous work

To date, no prospective studies have examined the effect of spaceflight on GM volume. However, one retrospective T2-weighted MRI study in 27 astronauts investigated intracranial and ocular irregularities on the basis of visual inspection. Except for deviations around the pituitary gland no other anomalies were reported [65]. The VBM8 pipeline that we used omitted the pituitary gland. Therefore, we were unable to evaluate any changes in that structure. The fact that no other effects of spaceflight were observed in the retrospective study could be due to insensitivity of visual inspection methods compared to the more objective, automated, and quantitative VBM approach. We have also recently published a retrospective MRI report showing decreases in astronauts' brain GM volume from pre- to post-flight [66]. Additionally, we observed small increases in GM volume in areas that control lower limbs. The regions in which there were GM decreases resemble those that we report as a function of HDBR, documenting similar outcomes between the two contexts.

The locations of the focal GM increases and decreases that we observed are very similar to the regions of tissue expansion and contraction respectively that Roberts and colleagues reported [21]. Furthermore, the GM increases and decreases that we observed after 7 days in HDBR overlap those reported by Li and colleagues after 30 days of HDBR [22]. Our results also corroborate several functional neuroimaging (fMRI) studies that have been conducted in HDBR. For example, a study of four subjects participating in 90 days of HDBR that combined fMRI with transcranial magnetic stimulation showed a post-HDBR increase in excitability of the motor cortex coding for leg musculature [19]. The increased excitability of the primary motor cortex was ascribed to relearning of leg control after long-term disuse during HDBR. This increased motor cortical excitability may also relate to the increased pre- and postcentral gyri GM volume that we observed. We previously reported on resting-state fMRI changes in the current sample of HDBR subjects [55]. We observed changes in functional connectivity strength between the left primary motor cortex with the right post-central gyrus/superior parietal lobule. This change in connectivity strength significantly correlated with balance performance changes from pre to post HDBR. Another resting-state fMRI study in 12 male participants revealed a decrease in signal amplitude from pre-HDBR to 72 hours post-HDBR in the left thalamus [20], which is a relay station for sensory and motor information. These functional MRI studies thus also support the idea that the gait and balance problems that astronauts experience post-flight could be related to central nervous system changes occurring in response to the body unloading experienced in microgravity.

The HDBR-induced sensorimotor problems that we observed are in line with previous studies that also reported deterioration of posture control and gait that showed at least partial recovery post HDBR [16, 17, 67–69]. We showed that sensorimotor performance deterioration was associated with structural brain changes. If our results would indeed translate to a microgravity environment, they suggest potential directions for developing countermeasures that would aid in ameliorating the adaptive effects of spaceflight on both the central nervous system as well as sensorimotor performance. Multiple terrestrial studies have shown the preventive and slowing effects of exercise on neural degeneration [70, 71]. In addition, aerobic plus resistance exercise in HDBR has already proved to be an effective countermeasure for the deconditioning effects of HDBR on the cardiovascular system and skeletal muscle [72]. Exercise interventions with increased axial body loading and potentially in combination with balance training might also prevent or mitigate HDBR induced changes in motor performance, brain function and brain structure.

The results of this study are not only relevant for astronauts, but also for individuals who are temporarily (e.g. after surgery or for pregnancy complications) or permanently (e.g. disabled) bedridden. They may further apply to elderly residents of nursing homes with reduced mobility [67]. The magnitude and extent of GM changes that we observed are quite striking, particularly given that the participants are young, healthy adults. Individuals who spend a substantial part of the day in a supine position for an extended period of time might be expected to develop similar GM and functional mobility changes as those observed in our HDBR subjects. Furthermore, with HDBR we observed decreases particularly in fronto-temporal regions, which are also most affected in aging [73]. Therefore, the effects of bed rest might interact with the effects of aging on fronto-temporal brain structure, and thus could lead to accelerated aging.

Our study is not without limitations. We looked at the effects of HDBR in 18 healthy male adults on brain structure and measures of mobility and postural equilibrium. The relatively small sample size may have precluded the detection of additional brain-behavior associations. Future studies should aim at obtaining larger samples and should include females as well as *males*. Even though we collected data at seven time points, the exact temporal relation between gray matter changes and HDBR remains unknown. The frontal GM decreases and posterior-parietal increases presented as early as 8.5 days in HDBR and continued to expand in size until 50.5 days in HDBR after which they seemed to plateau. Future studies should sample more frequently and earlier in bed rest in order to obtain insight into the exact time course of gray matter changes. In addition, we investigated a small spectrum of sensorimotor function. Including other measures of performance that are known to be impaired in astronauts post-flight may increase the generalizability of our results [74]. Additionally, MRI scans for the HDBR and control group were obtained using different scanners. It is possible that this may have introduced a bias, but there were no group differences in GM volume at baseline. Important strengths of our study are the inclusion of multiple assessments pre-, during, and post-HDBR that allowed us to investigate the temporal dynamics of brain changes and recovery. In addition, by directly comparing the changes over time in HDBR to control subjects we controlled for normative changes over time. To date, no other structural MRI HDBR studies have included more than two time points or a control group.

## 5. Conclusion

Seventy days of 6°-HDBR is associated with partially but not fully transient (after 12 days) volumetric GM changes in sensorimotor brain regions. Main areas of change include the primary motor cortex, somatosensory cortex, and the cerebellum. HDBR further leads to deterioration in functional mobility and balance. Such changes were all relative to a group of control subjects who did not show changes over a similar time course. GM changes in the precuneus cortex, precentral gyrus, and postcentral gyrus correlated positively with changes in balance. Our findings parallel the sensorimotor changes that astronauts experience with spaceflight. They furthermore support the use of HDBR as a spaceflight analog environment for studying the specific effects of axial body unloading on the sensorimotor effects observed with spaceflight. The observed gradual increases in posterior parietal GM and gradual decreases in frontal brain regions could reflect a combination of headward fluid shift related to increased intracranial pressure and fluid redistribution, as well as neuroplasticity. Future studies should aim to explore the mechanisms of and the countermeasures for the current HDBR-related GM changes. This is particularly important given NASA’s changing focus toward longer duration and more remote human exploration missions [75].

## Supporting information

S1 Table Overview of anatomical labels of local maxima of regions showing increases and decreases in gray matter volume with HDBR. (XLSX)
